# Editorial of Special Issue “Protein Post-Translational Modifications in Signal Transduction and Diseases”

**DOI:** 10.3390/ijms22052232

**Published:** 2021-02-24

**Authors:** Claudio D’Amore, Mauro Salvi

**Affiliations:** Department of Biomedical Sciences, University of Padova, 35131 Padova, Italy

The making of a protein is based on the combination of 20 different monomers (22 considering selenocysteine and pyrrolysine, the latest present only in some archaea and bacteria) giving the possibility of building a variety of structures from the simplest to the most complex, rigid or highly dynamic, and suited to carry out a wide range of structural and functional roles. However, proteins are required to adapt their structures to the needs of the organism and, in general, to any changes in the surrounding molecular environment, and are therefore subjected to continuous fine-tuning regulation. Post-translational modifications (PTMs) represent one of the most important mechanisms underlying this regulation. It is therefore not surprising that aberrant regulation of PTMs may be involved in different human diseases, including cancer, neurodegeneration, diabetes, and inflammatory diseases, and that the enzymes regulating their turnover are considered pharmacological targets of primary importance.

Among PTMs, phosphorylation is the most well-investigated and many writers (kinases), erasers (phosphatases), and readers (phosphor-binding domains) of this PTM have been thoroughly characterized; probably almost all biological events are in some manner under the control of a phosphorylation switch. However, despite all, a full picture of the role of phosphorylation is far from being reached—just to think that, to date, more than 200,000 phosphosites (according to PhosphositePlus, the most updated PTMs database [1]) have been identified—and ~95% of these are orphan, which means that the kinases responsible of their generation are unknown. Moreover, the functional role of these phosphosites, if any, is still to be deciphered. As if that were not enough, it is also well acknowledged that phosphorylation represents only one of a variety of PTMs, which include acetylation, methylation, glycation, ubiquitylation, and sumoylation, to name the best-known ones.

Our recently published Special Issue “Protein Post-translational Modifications in Signal Transduction and Diseases” includes six research articles and six review articles spanning a variety of PTMs and biological processes.

Protein methylation is a PTM that occurs at both lysine and arginine residues; this PTM has been mainly characterized in histone proteins and is associated with transcriptionally active chromatin. Khella et al. shed light on the mechanism by which Clr4 (a histone methyltransferase in *Schizosaccharomyces pombe*) may undergo auto-methylation and thus promoting self-activation. Furthermore, the authors proposed that Clr4 auto-methylation could act as a sensor of S-Adenosyl methionine cellular levels thus modulating heterochromatin formation according to the nutrient availability and the metabolic state of the cell [2].

The review proposed by Andrew and co-workers describes the main biological function of different PTMs affecting Histone H1 moving from lower eukaryotes to humans and highlighting their correlation with human diseases [3]. Histones are generally subjected to a variety of PTMs, with acetylation and methylation being the well-characterized ones, generating epigenetic marks by regulating DNA and transcription factor binding. Notably, protein methylation was traditionally considered a PTM specific for histone proteins, and only recently non-histone protein methylation is emerging as a widespread mechanism and the basis of the regulation of different biological processes [4].

Predicting PTM sites still remains one open challenge in biochemistry and biology. Aiming at improving the identification of potential disease-related phosphorylation patterns, Chen and colleagues developed a new in silico method for the prediction of kinase-specific phosphorylation, which has been dubbed GasPhos; the algorithm proposed by the authors could represent a useful tool to understand protein–kinase signaling in both physiological and pathological conditions [5].

An example of the crucial role exerted by phosphorylation in physiological conditions is reported by Syifa and colleagues. They ran a phosphoproteome analysis of mouse sperm during capacitation that led to the identification of 1050 new phosphorylated sites in 402 proteins potentially involved in sperm motility, outlining the role of GSK-3α and AKAP4 in mouse sperm capacitation [6].

Taking advantage of transgenic mice expressing biotinylated ubiquitin, Mercado-Gómez and collaborators profile the hepatic “ubiquitinome” in a model of liver fibrosis induced by CCl_4_ administration leading to a deeper comprehension of the potential role of protein ubiquitination in this disease [7].

It has been well established that nitric oxide (NO) plays a pivotal role in regulating the root development in plants; in their work, Niu et al. furnish experimental evidence that NO might induce adventitious rooting in cucumber through enhancing S-nitrosylation of a large variety of proteins. Moreover, functional analysis of nitrosylated proteins suggests that carbon and energy metabolism processes can be regulated by S-nitrosylation during root development [8].

Two papers of this special issue deal with PTMs in mitochondria. Even though PTMs in mitochondria have been often overlooked in the past, it has now been recognized that modifications of mitochondrial proteins represent a key mechanism in regulating organelle functions, including mitochondrial metabolic pathways, transport across the inner and outer membranes, fission, fusion, and degradation [9,10]. In their research article, Amer and Hebert-Chatelain, using a proximity-labelling technique, identified 21 proteins constituting the PKA intramitochondrial interactome and shed light on new potential functions exerted by the kinase in mitochondria [11]. Guerra-Castellano et al. in their review describe the PTMs regulating cytochrome c, a double-face protein–an essential component of the mitochondrial electron transport chain–and, at the same time, responsible for triggering apoptosis when released into the cytosol [12].

Not surprisingly, PTMs have also been identified in viral proteins. Kumar and colleagues take us on a journey into the world of the RNA virus, describing one by one all the most important PTMs identified in host and virus proteins regulating RNA virus replication [13].

Alpha-1-antitrypsin is an anti-inflammatory glycoprotein and a serine protease inhibitor produced by the liver and released into the bloodstream. Its genetic deficiency increases the risk of developing lung and liver disease. Lechowicz and colleagues in their review summarize the PTMs identified in this protein, and their functions, showing how an extracellular protein is also variably affected and regulated by PTMs [14].

Luiken’s group give us a detailed picture of the role of palmitoylation, a reversible modification consisting of the addition of palmitate to cysteine residues regulating protein targeting to membranes, in the insulin signaling pathways in the heart [15]. Moreover, the authors hypothesize the existence of a link between protein hyper-palmitoylation and insulin resistance and discuss the therapeutic potential of the regulation of palmitoylation enzymatic machinery.

The last review focuses on the role of lysine acetylation in Parkinson’s disease (PD), one of the most common neurodegenerative diseases. The authors show how acetylation of histone and non-histone proteins and the expression of the enzymes regulating their turnover (lysine acetyltransferases and deacetylases) are affected in PD patients and different PD models, discussing potential therapeutic interventions [16].

In the future, one important aspect that should be investigated more deeply is the crosstalk between different PTMs. Indeed, the same residue may be subjected to different mutually exclusive PTMs. For instance, it could happen that the same lysine may undergo methylation, acetylation, ubiquitylation, or sumoylation, and each of these may determine a different biological outcome [17,18]. In addition, proteins are subjected to several PTMs, that, when nearby, can affect each other by changing either protein folding or chemical surface [19]. To date, about 500,000 PTMs have been identified in 20,000 non-redundant proteins (PhosphositePlus database [1]), which means ~25 PTMs for protein. One of the biggest challenges for future research in this field is therefore deciphering the crosstalk between the various modifications generating a PTM-code, and how these PTM combinations can be read by specific effectors and transduced in a biological output.
